# Tackling neurodegeneration *in vitro* with omics: a path towards new targets and drugs

**DOI:** 10.3389/fnmol.2024.1414886

**Published:** 2024-06-17

**Authors:** Caterina Carraro, Jessica V. Montgomery, Julien Klimmt, Dominik Paquet, Joachim L. Schultze, Marc D. Beyer

**Affiliations:** ^1^Systems Medicine, Deutsches Zentrum für Neurodegenerative Erkrankungen e.V. (DZNE), Bonn, Germany; ^2^Genomics and Immunoregulation, Life & Medical Sciences (LIMES) Institute, University of Bonn, Bonn, Germany; ^3^Institute for Stroke and Dementia Research (ISD), University Hospital, LMU Munich, Munich, Germany; ^4^Munich Cluster for Systems Neurology (SyNergy), Munich, Germany; ^5^PRECISE, Platform for Single Cell Genomics and Epigenomics at the German Center for Neurodegenerative Diseases and the University of Bonn and West German Genome Center, Bonn, Germany; ^6^Immunogenomics & Neurodegeneration, Deutsches Zentrum für Neurodegenerative Erkrankungen e.V. (DZNE), Bonn, Germany

**Keywords:** neurodegeneration, drug discovery, drug repurposing, multi-omics, single-cell, *in vitro*, AD, PD

## Abstract

Drug discovery is a generally inefficient and capital-intensive process. For neurodegenerative diseases (NDDs), the development of novel therapeutics is particularly urgent considering the long list of late-stage drug candidate failures. Although our knowledge on the pathogenic mechanisms driving neurodegeneration is growing, additional efforts are required to achieve a better and ultimately complete understanding of the pathophysiological underpinnings of NDDs. Beyond the etiology of NDDs being heterogeneous and multifactorial, this process is further complicated by the fact that current experimental models only partially recapitulate the major phenotypes observed in humans. In such a scenario, multi-omic approaches have the potential to accelerate the identification of new or repurposed drugs against a multitude of the underlying mechanisms driving NDDs. One major advantage for the implementation of multi-omic approaches in the drug discovery process is that these overarching tools are able to disentangle disease states and model perturbations through the comprehensive characterization of distinct molecular layers (i.e., genome, transcriptome, proteome) up to a single-cell resolution. Because of recent advances increasing their affordability and scalability, the use of omics technologies to drive drug discovery is nascent, but rapidly expanding in the neuroscience field. Combined with increasingly advanced *in vitro* models, which particularly benefited from the introduction of human iPSCs, multi-omics are shaping a new paradigm in drug discovery for NDDs, from disease characterization to therapeutics prediction and experimental screening. In this review, we discuss examples, main advantages and open challenges in the use of multi-omic approaches for the *in vitro* discovery of targets and therapies against NDDs.

## 1 Introduction

Neurodegenerative diseases (NDDs) represent a growing societal and public health burden affecting millions of people worldwide with a projected increase to 3.3% of the population affected by 2060 (Erkkinen et al., 2018; Matthews et al., 2018). Briefly, NDDs are a heterogeneous group of neurological disorders characterized by the progressive loss of neurons either in the central or peripheral nervous systems (CNS, PNS). Beyond neuronal loss, NDDs result in the overall deterioration of both neuronal functions and synaptic plasticity, leading to a progressive reduction of patients’ behavioral, cognitive, sensory and motor abilities (Wilson et al., 2023).

As recently summarized, prevalent NDDs, such as Alzheimer’s and Parkinson’s disease (AD, PD), frontotemporal dementia (FTD) and vascular dementia (VaD), share a series of hallmark traits such as pathological protein aggregation, synaptic and neuronal network dysfunction, aberrant proteostasis, cytoskeletal abnormalities, altered energy metabolism, DNA and RNA defects, inflammation, and neuronal cell death (Wilson et al., 2023). While the root causes for each disease are potentially multi-factorial, heterogeneous, and disease-specific, these shared mechanisms account for the general dysfunction associated with the distinct NDDs (Wareham et al., 2022).

Despite the continuous scientific advances in our understanding of the pathophysiological traits of each NDD, the frequent failures of drug candidates in clinical trials even at advanced stage over the last few years, e.g., against AD, undermine the further engagement of pharmaceutical companies in developing new therapeutics against such disorders (Cummings et al., 2016). On the positive side, the advent of monoclonal antibodies, e.g., against β-amyloid (Aβ) to treat AD, spearheaded by the recent approval of Aducanumab and Lecanemab by the FDA, opens promising clinical scenarios counteracting pathological protein aggregation (Delrieu et al., 2024). Nevertheless, these agents pose a number of controversies related to the observed side effects, their overall accessibility and only partial efficacy in tackling advanced stages of the disease. Overall, possible reasons for the inefficient development of drugs against NDDs include (i) a lack of accepted consensus in biomarkers for patients’ stratification; (ii) the inappropriate choice of doses; (iii) an insufficient accounting for the stratification and heterogeneity of NDDs, as well as (iv) too late interventions (Kim et al., 2022).

Understanding the etiology of the major NDDs is further complicated by the diversity across different experimental models, which do not recapitulate in a univocal manner the major phenotypes observed in humans. While rodents and other model organisms represent an established and still useful option in NDDs research, numerous evidences attested their limited translatability into the human context (Dawson et al., 2018). A key example are microglia cells, for which the most advanced models are now relying on mouse xenotransplantation of either human pluripotent stem cells (hPSCs)-derived microglia or entire neuroimmune human organoids (Mancuso et al., 2019; Xu et al., 2020; Schafer et al., 2023). Such models have demonstrated to closely recapitulate human phenotypes by overcoming both the poor overlap between human and rodents as well as the cell state artifacts sometimes observed *in vitro* (Zhang et al., 2023). Nevertheless, xenotransplantation is still animal-based, laborious and only partly reproducible, thus it barely complies with classical requirements for early drug discovery (Hughes et al., 2011).

With the advent of highly sophisticated 3D *in vitro* models we are entering a new phase of drug discovery (Klimmt et al., 2020; Whitehouse et al., 2023). Earlier, neuron-centric *in vitro* cell culture models failed to capture the involvement of glial cells and therefore could not model important parts of NDDs pathophysiology such as neuroinflammation. More recent co-culture systems including astrocytes and microglia better approximate CNS cell-type heterogeneity and thus increase their suitability for disease modeling. Further advancing models from 2D monocultures to heterogeneous co-culture systems, up to the highly-structured 3D organoids has allowed to achieve even more complex disease phenotypes *in vitro* (Klimmt et al., 2020). In addition, significant advances have been achieved with the introduction of human induced pluripotent stem cells (hiPSCs) (Penney et al., 2020). Human iPSCs allow to generate neuronal and glial cell types with a well-defined, patient-derived genetic background (Li and Shi, 2020; Dannert et al., 2023). This is of particular interest to further model genetic variability in the context of personalized drug discovery. However, further improvement of iPSC-based models is required as their early developmental state does not yet optimally recapitulate the environment in which late-onset diseases like AD develop. Direct conversion of human fibroblasts into neurons (induced neurons, iNs) while bypassing their reprogramming into iPSCs followed by differentiation, was demonstrated to better preserve aging phenotypes *in vitro* (Mertens et al., 2015), but these models are still constricted, e.g., by limitations in cell sources and accessibility to gene editing. The establishment of more mature, scalable model systems might thus be a prerequisite for further improving reproducibility, controllability, and efficiency of drug discovery (Okano and Morimoto, 2022).

In light of these advancements, there is a pressing need to revise the overall drug discovery and repurposing strategy while accounting for the heterogeneous, multi-factorial, and multi-target nature of NDDs. Along these lines, the combination of systems medicine and novel pharmacological approaches would enable scientists to complement reductionist approaches to capture major aspects of the disease complexity (Balusu et al., 2023). As such, tools are required that can disentangle disease states and model perturbations through the comprehensive characterization of distinct molecular layers up to the single-cell resolution. Here, omics approaches have the potential to capture cellular information on a global level for distinct molecular layers (i.e., genome, transcriptome, proteome) (Hampel et al., 2021; Arenas, 2022). Because of their increasing affordability, the use of omics technologies to drive drug discovery, while still a relatively young development in the neuroscience field, is now spreading (Van de Sande et al., 2023). With transcriptomic analyses leading the field, these high-dimensional approaches have been increasingly used to accelerate the early discovery and repurposing of drug candidates when coupled to *in vitro* perturbations (Carraro et al., 2022), for the pharmacological and toxicological evaluation of *in vivo* treatments (Nguyen et al., 2022) as well as for monitoring and systemic investigation of disease susceptibilities in clinical cohorts (Zielinski et al., 2021).

Unimodal single-cell omics measurements have revolutionized our knowledge on the cellular heterogeneity of organs and tissues, up to the definition of human cell atlases, informing us on the cellular mechanisms of plasticity in health and disease (Vandereyken et al., 2023). Nevertheless, multi-omics approaches, i.e., the combination of distinct omics layers of information such as the genome, epigenome, transcriptome, proteome, lipidome, and metabolome have improved our way to interpret cellular processes as a whole even more profoundly. An integrated multi-omics perspective on pathological states offers new avenues to increase our understanding of disease trajectories up to the possible definition of endotypes. Unveiling the gene regulatory networks underlying NDDs with single-cell multi-omics provides scientists with a holistic view on the possible contribution of the different molecular layers and how these result in the observed cellular phenotypes (Vandereyken et al., 2023). Different protocols and technologies have been developed in the last decade to enable scientists to assess different omics information, from bulk to single-cell resolution, and many options are now commercially available (Baysoy et al., 2023). In addition, more recent options finally allow for the simultaneous detection of multiple omics measurement from the same single-cell (multi-modal omics) (Zhu et al., 2020). In parallel, huge efforts have allowed the establishment of robust bioinformatics pipelines to ensure the integration of multiple omics information, so as to speed up the biological interpretation of such high-dimensional datasets including recent pipelines in R and Python (Hao et al., 2021; Gayoso et al., 2022; Vandereyken et al., 2023). Finally, distinct computational frameworks have been developed to efficiently tackle the technical and biological difficulties of integrating and harmonizing the information obtained from distinct omics measurements to obtain a unified landscape of the cellular regulatory networks underlying disease (Miao et al., 2021).

In this review, we will discuss the main achievements and open challenges in the use of multi-omics for the characterization of NDDs and the discovery of therapeutics against neurodegeneration, with particular attention to AD and PD as the most prevalent and extensively characterized NDDs. While both have their disease specifics, the approach itself is generalizable to most NDDs. In detail, we will focus on the role of omics approaches in the early phases of drug discovery, from the evaluation of *in vitro* models as a fundamental step for efficient drug screening, through the bioinformatics analysis of omics data for the identification of biomarkers and druggable pathways in NDDs, up to the most recent examples on the use of omics to identify novel drug candidates. To conclude, we will discuss some general requirements and key steps to optimize the design of omics-based drug discovery and repurposing experiments.

## 2 Benchmarking *in vitro* models of neurodegeneration with multi-omics

*In vitro* models for early drug discovery should efficiently recapitulate the main pathophysiological phenotypes observed in humans while retaining critical features such as reproducibility and scalability (Whitehouse et al., 2023). Over the last years, multiple systems have been proposed to study NDDs (Slanzi et al., 2020; Qian and Tcw, 2021). As already mentioned, these different *in vitro* platforms have particularly benefited from the introduction of hiPSC-derived cell cultures (Paquet et al., 2016). Different hiPSCs-based models with increasing spatial complexity are available, spanning from 2D to 3D, i.e., based on the use of artificial matrix scaffolds or coupled with microfluidics devices (Park et al., 2018), up to the highly structured organoid systems (Lancaster et al., 2013; Bershteyn et al., 2017; Chiaradia and Lancaster, 2020), and are increasingly employed to study brain development and disease.

**Figure 1:**
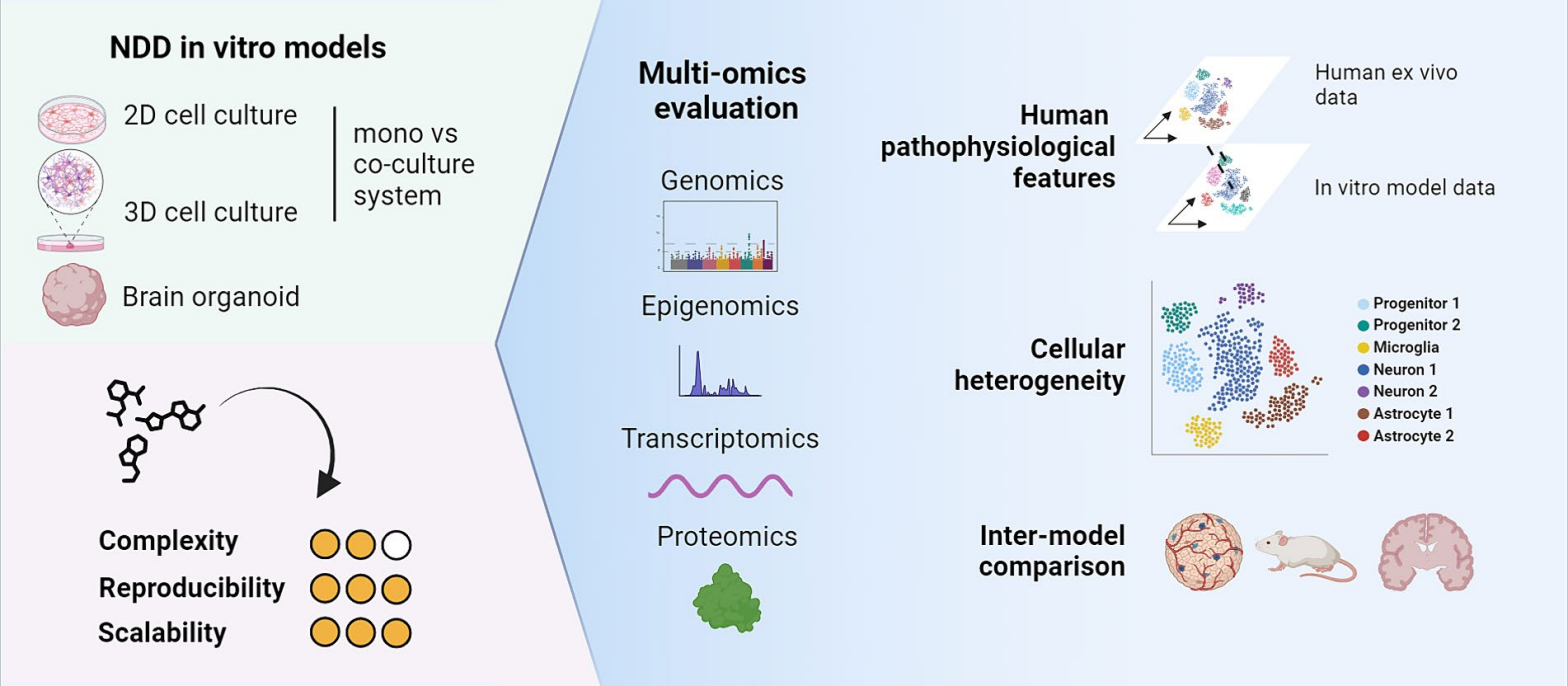
Multi-omics technologies can be useful tools to characterize cellular heterogeneity and maturity of *in vitro* models. Key aspects to consider are their similarity to human characteristics and their suitability as *in vitro* models for drug and target discovery. Created with BioRender.com.

This diversity poses now the challenge to choose the most suited *in vitro* system when establishing a platform for further drug activity assessments. As such, omics technologies can be powerful tools to evaluate *in vitro* systems for their suitability as screening systems for drug and target discovery (Figure 1). In this section, we will describe the value of omics technologies for the characterization of *in vitro* models of NDDs (particularly AD and PD). We will showcase how these technologies helped to evaluate different models in terms of cellular heterogeneity and similarity to human phenotypes, concluding with some remarks on the advantages and challenges for their proper implementation in future drug discovery pipelines (Table 1).

**Table 1:** Examples for the use of multi-omics for the characterization of *in vitro* NDD models.

NDD model	Applied omics technology	Characterized features	Model limitations
2D hiPSCs monocultures Yagi et al. (2011), Israel et al. (2012), Bardy et al. (2016), Handel et al. (2016), Lin et al. (2018), Lang et al. (2019), Burke et al. (2020), Fernandes et al. (2020), Badanjak et al. (2021), Cardo et al. (2023), Dolan et al. (2023)	Microarrays, bulk and single cell RNA-seq and ATAC-seq	Phenotype maturation, pathological features, impact of distinct genotypes and donor genetic background, similarity with human material, reproducibility for HT screens, epigenomic characterization of cell states	Mostly focused on neuronal characterization, no spatial complexity, poor milieu heterogeneity and consequently limited representation of *in vivo* pathological burden
2D hiPSCs co-cultures Lin et al. (2018)	Bulk RNA-seq	Phenotype maturation, cell types’ and states’ heterogeneity, pathological features, glial-neuronal crosstalk, effect of engineered genetic background	No spatial complexity, limited representation of *in vivo* pathological burden
3D hiPSCs-derived co-cultures (neurons, astrocytes, microglia) Papadimitriou et al. (2018), Jorfi et al. (2023), Cuní-López et al. (2024)	Bulk RNA-seq, single cell RNA-seq	cell plasticity, phenotype maturation, cell types’ and states’ heterogeneity, spatial complexity, enhanced pathological features, enhanced glial-neuronal crosstalk, *ex vivo* phenotype comparisons, neuroimmune axis characterization	Use of exogenous scaffold matrix, major neuronal focus, often not all glial cell types included, heterogeneous modeling of human pathological features
Cerebral organoids Zhao et al. (2020), Chen et al. (2021), Kim et al. (2021), Mohamed et al. (2021), Zagare et al. (2022), Patikas et al. (2023), Vanova et al. (2023)	Single cell RNA-seq	Increased spatial patterning, phenotypes in developmental modeling, glial-neuronal crosstalk, *ex vivo* phenotype comparisons, neuroimmune axis characterization, phenotype maturation, characterization of genetic contribution to observed pathology	Limited reproducibility and scalability, better approximation of developmental rather than NDD phenotypes and maturation
Xenotransplated microglia McQuade and Blurton-Jones (2019), Claes et al. (2021)	Single-cell RNA-seq	Evaluate maturation in presence of *in vivo* milieu, *ex vivo* phenotype comparisons, neuroimmune axis characterization, phenotype maturation, characterization of genetic contribution to observed pathology	Limited reproducibility and low scalability, still rely on *in vivo* models, heterogeneous modeling of human pathological features

### 2.1 Evaluation of *in vitro* models of AD using omics approaches

AD is the most common NDD but its pathophysiological complexity is still not fully elucidated. A major hallmark of this disease is the formation of amyloid beta plaques and tangles of hyperphosphorylated tau protein, associated with neuroinflammation and synaptic dysfunction, ultimately leading to neuronal cell death (Wilson et al., 2023).

The advent of new technologies mentioned above helped with widening our knowledge on possible mechanisms underlying AD, enabling scientists to advance the complexity of available *in vitro* systems to model key aspects of the underlying pathology. Numerous *in vitro* systems with increasing complexity have been reported to model the main molecular signs of AD. Among the first available options, neuron-focused 2D iPSC systems were developed (Yagi et al., 2011; Israel et al., 2012; Bardy et al., 2016; Handel et al., 2016; Lin et al., 2018; Burke et al., 2020). Among others, the one reported by Kondo et al. (2017) was used to test the ability of different compounds to clear amyloid beta *in vitro*. Transcriptomics enabled the molecular phenotyping of iPSC-derived *in vitro* models across different CNS cell types, providing a useful comparison to human *ex vivo* CNS profiles in health and disease states (Table 1). As an example, Lin et al. (2018). analyzed the transcriptome of iPSC-derived neurons, astrocytes and microglia-like cells bearing an APOE4 genotype, unveiling impaired synaptic function, lipid metabolism and immune response compared to isogenic APOE3 cells.

While extremely useful and widely adopted by the community, 2D mono and co-culture models can only partly recapitulate the multiplicity of phenotypes and molecular processes contributing to AD, such as concurrent amyloidosis and tau pathology. Moreover, the low spatial complexity of these cultures does not allow to inspect more complex inter-cellular and cell-matrix interactions, all key aspects to consider when evaluating the potential of novel therapeutic agents. In this direction, a step forward was achieved with the introduction of 3D mono- and co-culture systems, from spheroids to highly-structured organoids. In the last decade, several 3D models were developed that replicate distinct features of AD such as amyloid deposition, tau burden and more complex cellular interactions (Choi et al., 2014; Raja et al., 2016; Gonzalez et al., 2018). In this scenario, transcriptomics was increasingly employed to characterize cell type and state-specific gene expression profiles, depicting a highly granular range of cellular phenotypes in such 3D models. Different engineering approaches have been also used to generate 3D *in vitro* culture systems that span from microfluidics devices to hydrogels of different composition (Park et al., 2015; Papadimitriou et al., 2018; Jorfi et al., 2023; Cuní-López et al., 2024). Papadimitriou and colleagues proposed a 3D culture of neural stem cells or primary cortical astrocytes embedded in a biohybrid hydrogel scaffold after treatment with Aβ42 peptide as a platform to study AD (Papadimitriou et al., 2018). A whole-transcriptome assessment revealed different cell-type-specific gene expression signatures proving that cortical alterations are present in these conditions. This analysis also confirmed the presence of more complex neuronal networks in culture settings of higher spatial complexity. Single-cell transcriptomics allows in addition to finely describe the advanced range of represented cellular phenotypes. As an example, Cuní-López et al. (2024) used a tissue engineering approach to create 2D and 3D co-cultures of monocyte-derived microglia cells (MDMis) and immortalized neuronal progenitors. They tailored this *in vitro* platform to study Alzheimer’s disease by generating their MDMis both from AD patients with different stages of the illness and from healthy controls. A single-cell RNA-seq (scRNA-seq) analysis then showed a greater heterogeneity of cell populations in the three dimensional culture settings when compared with the 2D counterpart. Moreover, in the AD 3D co-cultures, a reduced contact between microglia and neuronal cells after immunohistochemical surface reconstruction, and a donor-specific cytokine response pattern to dasatinib treatment were observed. A even higher diversity of profiles was investigated by Jorfi et al. (2023) who also used single-cell transcriptomics to evaluate their novel 3D stem cell-derived microfluidic system which included not only CNS-related cell types such as immortalized neurons and astrocytes, and microglia, but also incorporated peripheral immune cells to depict a finer grained picture of the neuroimmune interaction between glial cells and infiltrating T cells in the AD brain. Transcriptomics has been widely employed to characterize *in vitro* AD models of higher spatial complexity in terms of cellular heterogeneity and functional profile, with the aim to clarify how closely such models could represent pathogenic processes observed in humans (Zhao et al., 2020; Chen et al., 2021). As an example, Chen and colleagues leveraged scRNA-seq to explore the functional pathways involved in the response of neurons and astrocytes to blood brain barrier (BBB) leakage, simulated *in vitro* by treating cortical organoids derived from iPSC cells of AD patients with serum from healthy donors (Chen et al., 2021). More recently, the advent of spatial transcriptomic technologies offers yet novel tools to map phenotypes and cellular interactions in tissues and 3D *in vitro* models. Interestingly, Vanova et al. (2023) showed how iPSC-derived brain organoids bearing PSEN1 and PSEN2 gene mutations can mimic Alzheimer’s disease-like pathology. Remarkably, scRNA-seq highlighted a defective development of specific progenitor cells and the absence of an organized structure in AD cerebral organoids (Vanova et al., 2023).

Furthermore, the possibility to assess multiple omics modalities jointly, up to single-cell resolution, allowed to obtain a more detailed picture of the heterogeneity of cell types and states. Dolan et al. (2023) leveraged both single-cell transcriptomics and chromatin accessibility analysis to characterize their *in vitro* model of human stem cell differentiated microglia (iMGL). The study assessed the iMGL transcriptomic profile at the single-cell level upon exposure to different substrates present in the microenvironment of an AD brain, i.e., synaptosomes, myelin debris, apoptotic neurons or amyloid-beta fibrils. The work identified different microglia cell states, including a cluster of disease associated microglia (DAM), a cell population accumulating around amyloid plaques first reported in a murine model of AD (Keren-Shaul et al., 2017) and later identified in multiple *in vitro* and *ex vivo* settings in murine models and, albeit with other distinctive features, in humans.

Concerning genetic alterations, genome-wide association studies (GWAS) unveiled numerous genetic risk factors associated with AD, such as APOE4 and TREM2 (R47H), inspiring the development of models aimed to better depict the genetic predisposition for AD *in vitro*, often obtained through genetic engineering (McQuade and Blurton-Jones, 2019; Claes et al., 2021). Besides characterization of the genetic underpinnings driving AD development, transcriptome analyses allow for the definition of regulatory dynamics in target cell populations and can potentially unveil additional mechanisms underlying NDDs. As one example for this, Claes et al. (2021) investigated the role of a TREM2 R47H mutation on human iPSC-derived microglia xenotransplanted in a murine AD model, demonstrating a transcriptome similar to human atherosclerotic foam cells and only a limited reactivity toward amyloid plaques. Such phenotype was also described by McQuade and Blurton-Jones (2019), who showed by scRNA-seq that TREM2-knockout iPSC-derived human microglia xenotransplanted into a murine model failed to activate in response to amyloid-beta plaques. Altogether, these studies showed that it is crucial to not only use functional assays but complement this with transcriptomic analysis of microglia to unravel the complex function of TREM2 and its genetic variants in microglia, especially in AD pathology.

Overall, omics technologies not only allowed the exploration of a wider spectrum of possible biological processes relevant to AD pathology but also helped to evaluate to which extent such processes could be recapitulated in different models, especially *in vitro*. This has enabled researchers to investigate the effect of different genetic risk factors and other phenotypic AD features, not only for neurons but also glial cells in progressively more advanced 3D co-culture models. These advances will be of great advantage for the reliable early-phase screening of novel potential drug candidates against AD and their mechanism of action.

### 2.2 Omics evaluation of *in vitro* models of PD

PD is a neurodegenerative disease with a multifactorial nature, characterized by symptoms spanning from resting tremor to neuropsychiatric manifestations for which the most commonly available symptomatic treatment is levodopa (Jankovic and Tan, 2020). A pathognomonic hallmark underlying PD is the accumulation of α-synuclein in dopaminergic neurons, leading to cell dysfunction and neuronal death in the substantia nigra (Krach et al., 2020).

Several key steps have been undertaken over the last decade to model PD *in vitro*. As different mutations and copy number variations are associated with PD, iPSC-derived models were developed reflecting this genetic complexity to investigate the genetic bases of the disease (Sim et al., 2020; Virdi et al., 2022; Xu P. et al., 2022). Most of these culture approaches focus on the generation of dopaminergic neurons that are either genetically modified or directly derived from patients. Lang and colleagues opted for the second approach, producing iPSC-derived dopamine neurons from three different carriers of the GBA-N370S mutation and then applied scRNA-seq for transcriptome profiling (Lang et al., 2019). Remarkably, their assessment highlighted the higher endoplasmic reticulum stress in two of the PD donors-derived lines when compared with healthy controls and a downregulation of a set of genes dependent on HDAC4 expression, later designated as a possible target for treatment. Furthermore, the incubation of these cultures with the HDAC4 allosteric inhibitor tasquinimod led to a reduction of α-synuclein release. In addition, this transcriptomics analysis refined the diagnosis for one of the donors as a progressive supranuclear palsy patient. Single-cell transcriptomics have also been employed to describe cell specific responses and transcriptional dynamics upon perturbation, as reported for iPSC-derived dopaminergic neuron cultures and midbrain organoids exposed to rotenone, a pesticide known to induce PD-like symptoms (Fernandes et al., 2020; Patikas et al., 2023). The study highlighted the presence of distinct cell subpopulations showing different transcriptomic responses to treatment and described potential underlying biological pathways. In an analogous fashion, Cardo et al., treated iPSC-derived midbrain neurons with 1-Methyl-4-phenyl-1,2,5,6-tetrahydropyridine (MPTP), a compound known to cause selective cellular damage mimicking PD-related cell toxicity effects. The team used scRNA-seq to follow the development in culture of the iPSC-derived, CRISPR-Cas9 edited tracer line among four different timepoints to evaluate the model’s maturity and determine the best setting for MPTP treatment. Insights on cell-type-specific transcriptomic changes upon compound administration were also gathered (Cardo et al., 2023). Additional mechanisms like neuroinflammation are known to contribute to PD pathological processes, and to further explore this avenue other *in vitro* models containing also non-neuronal cells are now available. A recently published work leveraged transcriptomic analysis of both iPSC- and postmortem-derived microglia for the construction of a single-cell midbrain *in vitro* atlas depicting the role of microglia in sporadic PD (Badanjak et al., 2021).

Three-dimensional culture systems are cell-heterogeneous platforms whose spatial complexity is well-suited to model PD for drug discovery purposes. In this direction, studies have generated midbrain organoids from individuals carrying a triplication of the SNCA gene to reproduce key aspects of the PD pathology *in vitro* (Mohamed et al., 2021; Zagare et al., 2022; Patikas et al., 2023). Their characterization by scRNA-seq allowed to explore the heterogeneity of the represented cellular profiles compared to other *in vivo* and *in vitro* models. This comparative analysis unveiled shared consensus features across the different models, highlighting the potential of single-cell tools to evaluate and score cellular and phenotypic complexity across disease models. The use of such tools enabled also the characterization of other organoid models of PD, like the one published by Zagare et al. (2022), who generated midbrain cultures from iPSC lines edited to carry the LRRK2-p.Gly2019Ser mutation. The transcriptomic profiling carried out by this group allowed for the evaluation of differences between mutated and wild-type samples both in cellular proportions and gene expression, indicating an altered developmental process in the organoids carrying the PD alteration. hESC-derived midbrain-like organoids were also used as a source of neural progenitors for transplantation into a hemiparkinsonian rat model to probe a new regenerative medicine approach for treatment. Both bulk and single-cell analysis were useful to determine the 3D culture setting as the most appropriate supply of selected progenitors for the procedure, when compared with 2D-derived NSCs, resulting in amelioration of performance in a battery of tests measuring forelimb akinesia (Kim et al., 2021). Overall, omics technologies have shown great promise to support the development of relevant *in vitro* models phenocopying Parkinson’s disease pathology. In particular, the integration of 2D and 3D models with *ex vivo* data offered the possibility to highlight consensus features, inter-model similarity as well as limitations such as immature phenotypes and model artifacts.

To conclude, multi-omics represent powerful tools to evaluate the cellular heterogeneity and complexity of *in vitro* models, key aspects to be considered for the selection of the most suited drug discovery system for NDDs (Sharma et al., 2020). Single-cell omics can convey a high degree of granularity, difficult to achieve with other technologies, enhanced by the recent possibility to investigate spatial and longitudinal information. Nevertheless, such fine-grained pictures often vary across different models and datasets, both in terms of distinct phenotypes as well as their annotation, and efforts are needed to generate consensus information (Paolicelli et al., 2022), in order to eventually identify the most convenient and reliable scaffolds for the early identification of novel therapeutics *in vitro*.

## 3 Omics in drug discovery and repurposing for neurodegenerative diseases

**Figure 2:**
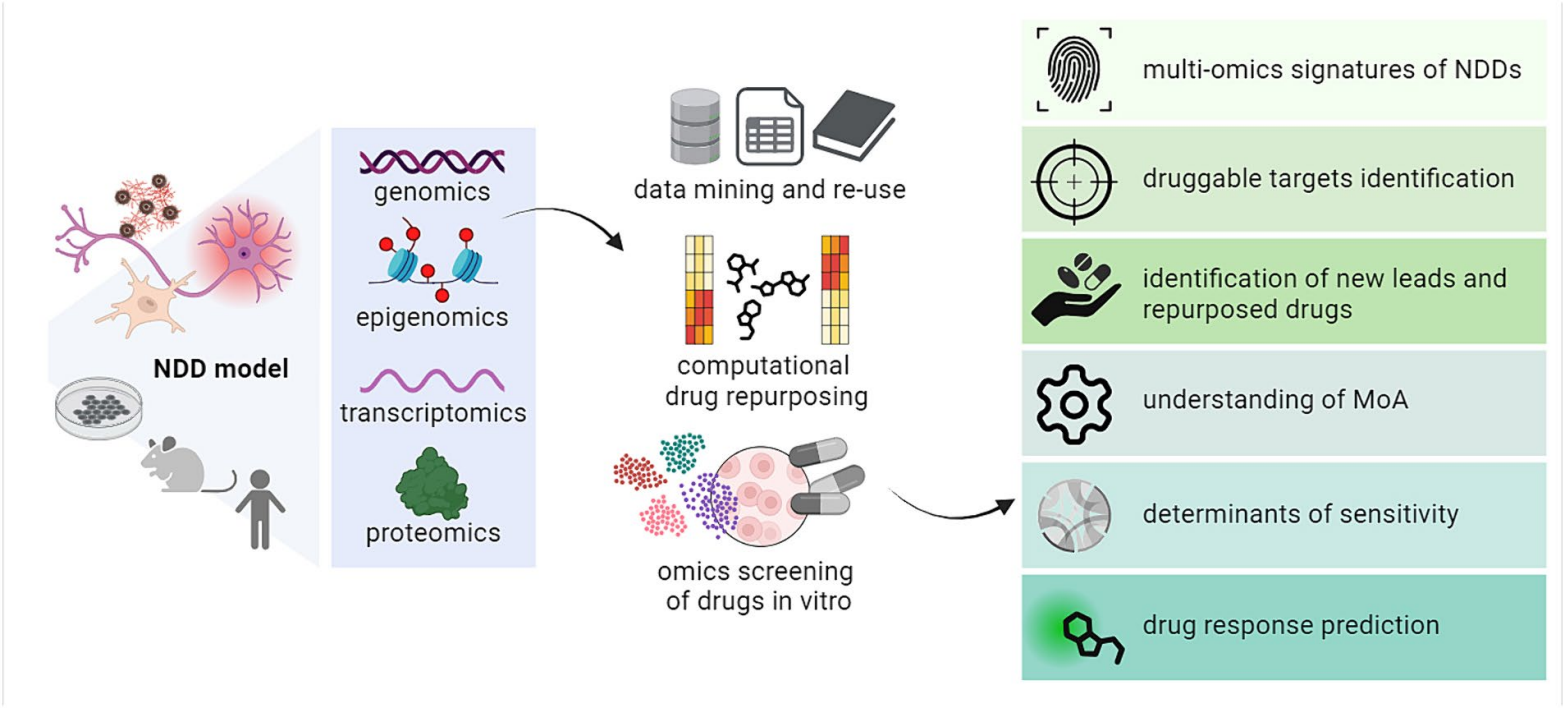
Overview of the various applications of multi-omics in drug discovery and repurposing against targets in NDDs. MoA, mechanism of action. Created with BioRender.com.

Recent years have seen a rise in the use of omics and computational approaches to fuel drug discovery. Different studies have demonstrated how to exploit omics to advance the identification and characterization of novel or repurposed therapeutics, from the study of their activity, mechanisms of action, and pharmacodynamics up to the investigation of cellular responses and determinants of susceptibility (Van de Sande et al., 2023). More recently, many reports have shed light on the potential for combining multi-omics with other computational tools to foster the discovery of drug candidates against targets in different NDDs (Aerqin et al., 2022). In this section, we will summarize the main findings and examples related to (i) the computational mining and re-use of existing omics and other high-content datasets to identify novel druggable targets in neurodegeneration as well as to build disease signatures; (ii) some of the available approaches for omics-based repurposing of drugs, with key examples of their efficient application; (iii) the use and establishment of omics-based experimental workflows for the screening and characterization of small molecules against known and unknown target structures in NDDs (Figure 2).

### 3.1 Data mining and re-use to identify new targets and disease signatures in NDDs

In the last decade, the scientific community has produced a considerable amount of multi-omics data describing cellular processes and phenotypes in health and neurodegeneration (Balusu et al., 2023; O’Connor et al., 2023). These bulk and single-cell datasets, obtained from different complementary models of NDDs, have provided us with a holistic view of the complex cellular dynamics regulating different neurological processes at high resolution (Piwecka et al., 2023). These converged in the generation of high-dimensional atlases describing the cellular heterogeneity of different areas of the CNS in mouse and human as well as in brain organoids (Kanton et al., 2019; Agarwal et al., 2020; Rood et al., 2022; Zhou et al., 2022; Mathys et al., 2023). This enormous amount of data, becoming increasingly accessible to the research community, calls for further mining and mindful re-use to generate additional knowledge also through cross-dataset integration (O’Connor et al., 2023). Such a perspective sounds particularly attractive in the drug discovery context as we could potentially perform pre-assessment of a given patient’s vulnerabilities and project disease trajectories through integration of multiple existing datasets.

Different databases and repositories have been established and curated in the last few years to allow scientists to systematically access multi-omics and other genomic and phenotypic datasets (Table 2) (Abdullatef and Farina, 2023; O’Connor et al., 2023). Among the most widely known are Gene Expression Omnibus (GEO) and the European Genome Archive (EGA) for NGS data, BioImage Archive (BIA) for imaging and microscopy-based spatial omics data, as well as PRoteomics IDEntifications (PRIDE) for proteomics datasets, plus many others, including some for lipidomics and metabolomics data (Martens et al., 2005; Barrett et al., 2013; Lappalainen et al., 2015; Hartley et al., 2022). Interestingly, also resources for the specific deposition of neurological datasets exist, such as the NIH BRAIN Initiative Cell Census Network (BICCN), the Mount Sinai Brain Bank and ROSMAP dataset, the Allen Brain Map and the Alzheimer’s Disease Neuroimaging Initiative (ADNI), Synapse AD knowledge portal, Brain-CODE and the National Cell Repository for Alzheimer’s Disease (NCRAD), which offer access to catalogs of biobanked specimens and the associated metadata (Petersen et al., 2010; Bennett et al., 2018; Wang et al., 2018, 2020; Hawrylycz et al., 2023; National Institute on Aging, n.d.).

**Table 2:** Overview of the main general and neuroscience-related databases and repositories for the deposition and access of multi-omics, imaging, clinical and other phenotypic data.

Name	Data type	Reference
Allen Brain Map	Gene expression, spatial mapping, cell atlassing	http://www.brain-map.org, Wang et al. (2020)
National Cell Repository for Alzheimer’s Disease (NCRAD)	Biobanking specimen and metadata catalogues	https://ncrad.iu.edu/
NIH BRAIN Initiative Cell Census Network (BICCN)	Transcriptomics, epigenomics, spatial, morphology, imaging, multimodal	https://biccn.org/, Hawrylycz et al. (2023)
Mount Sinai Brain Bank	Genomics, transcriptomics, proteomics, imaging, neuropsychological assessments	deposited at https://synapse.org/, Wang et al. (2018)
Alzheimer’s Disease Neuroimaging Initiative (ADNI)	MRI and PET images, genetics, cognitive tests, CSF and blood biomarkers as predictors	https://adni.loni.usc.edu/, Petersen et al. (2010)
Brain-CODE	Platform for data management and sharing. From clinical assessment data to neuroimaging, PET, MRI	https://www.braincode.ca/
Synapse/AD knowledge portal	Genomics, transcriptomics, metabolomics, proteomics	https://adknowledgeportal.synapse.org/
BioImage archive (BIA)	Imaging	https://www.ebi.ac.uk/bioimage-archive/, Hartley et al. (2022)
Gene Expression Omnibus (GEO)	Gene expression	https://www.ncbi.nlm.nih.gov/geo/, Barrett et al. (2013)
European Genome Archive (EGA)	Genetic/genomic, phenotypic, clinical	https://ega-archive.org/, Lappalainen et al. (2015)
PRoteomics IDEntifications (PRIDE)	Proteomics	https://www.ebi.ac.uk/pride/, Martens et al. (2005)

It has been recently demonstrated that the multi-omics information from published datasets can drive the discovery of new potential NDD targets, biomarkers and disease signatures (Hampel et al., 2021; Aerqin et al., 2022). This will be instrumental in the future for the downstream computational repurposing of chemical agents. As an example, publicly available GWAS, e- and pQTLs datasets from blood, CSF and brain tissue biopsies have been integrated to achieve a more comprehensive overview of potential causal genes and targets for intervention in PD (Gu et al., 2023). In another examples, multi-omics analyses of *ex vivo* and *in vitro* disease models further elucidated the contribution of dysregulated lipid and amino-acid metabolism pathways to PD pathogenesis and highlighted PD-associated gene signatures through the combination of transcriptomics, epigenomics and metabolomics (Lee A. J. et al., 2023; Zagare et al., 2023). Numerous studies have described disease phenotypes of different CNS cell-types in AD resulting in the definition of genomic determinants and transcriptomic signatures of the disease, further complemented by *in vitro* modeling as described above (Grubman et al., 2019, 2021; Xicota et al., 2019; Mathys et al., 2023). In addition, single-cell resolution and spatial approaches have enabled the identification of cell-type specific signatures of NDDs, as reported for a subset of dopaminergic neurons in the substantia nigra pars compacta (SNpc) of PD patients (Kamath et al., 2022), or for glial subsets within idiopathic PD patients’ midbrain (Smajić et al., 2022). Similarly, as an example of non-neuronal glial cells deeply implicated in disease pathology, DAM microglia represent a druggable glial phenotype identified by scRNA-seq as enriched in AD with a potential key role in the disease pathogenesis (Deczkowska et al., 2018; Grubman et al., 2021). Numerous studies have performed metabolome analysis for the definition of AD and PD biomarkers and the identification of potentially druggable targets (Lista et al., 2023; Yin et al., 2023). Recently, Liu et al. (2024) conducted a metabolome-wide association study in AD (MWAS) which led to the identification of fourteen metabolites showing an association with AD risk. de Lope et al. (2024) identified PD-associated plasma metabolome alterations, particularly in xanthine metabolism, in line with other independent studies on transcriptomics data. Lastly, proteomics investigations have been increasingly recognized as pivotal for understanding the complex protein pathophysiology underlying NDDs (Rayaprolu et al., 2021). As an example, Karayel et al. (2022) recently developed a scalable, sensitive, and reproducible proteomics protocol for PD biomarker discovery. Further, Sung et al. (2023) propose the combined usage of brain, CSF, and plasma proteomics to identify markers for sporadic and genetically defined AD.

Overall, newly generated data combined with the re-use and integration of existing multi-omics datasets, especially from human cohorts, represents a powerful resource to pre-identify disease trajectories and signatures useful for the downstream, patient-tailored identification of novel targets and therapeutics against NDDs. Joint and comprehensive efforts are needed to achieve a transversal consensus derived from the existing high-dimensional NDD multi-omics datasets, spanning across different models and clinical cohorts.

### 3.2 Computational strategies for omics-based drug repurposing

The concept of drug repurposing refers to the renewed application of established drugs beyond their original therapeutic indication (Pushpakom et al., 2019). The repurposing of therapeutics offers an accelerated route in drug development by excluding the need for further preclinical and toxicological testing as well as early clinical trials up to phase II trials. For this reason, drug repurposing represents an appealing opportunity to tackle NDDs, especially in light of the decreased investments of pharmaceutical companies in this high-risk therapeutic area (Kakoti et al., 2022).

Different approaches have been reported to identify potential candidates to be repurposed against different NDDs, based either on Delphi consensus or computational strategies (Ballard et al., 2020). Delphi consensus converges the knowledge of experts in the pathological field of interest who scrutinize the literature to comprehensively and systematically identify potential candidates for repurposing, as reported for rho kinase inhibitors (Fasudil), acetylcholinesterase inhibitors (Phenserine) and antiviral drugs against AD during the 2018–2019 Delphi process (Ballard et al., 2020). While such an approach can potentially unravel promising hits for further development (Corbett et al., 2012), the biased shortlisting of potential candidates tends to neglect the dynamic nature of drug treatments, which generally not only induces a direct perturbation of the intended pathways and targets, but rather a more comprehensive cellular response even directly or indirectly (e.g., off-targets) (Padhi and Govindaraju, 2022).

Computational approaches can circumvent this by considering the multi-target nature of most NDDs as well as the cellular heterogeneity of disease states (Paranjpe et al., 2019). One example are biophysical methods, including structural, ligand-based and molecular docking strategies for the modeling of drug-target interactions *in silico* (Paranjpe et al., 2019). Still, in the majority of cases, these approaches start from *a priori* knowledge on potential targets to be investigated for a given disease, leading to a literature-based inspection of candidates for further repurposing. Other agnostic options rely on the generation of clinical signatures from the analysis of patients’ medical histories and, in general, electronic medical records (Paik et al., 2015). While potentially very useful, the limited accessibility of sensitive clinical data represents a major obstacle to the widespread use of such approaches (Gehrmann et al., 2023).

As a promising alternative, transcriptome-based approaches primarily rely on the identification of drug hits capable of reversing specific disease signatures (Shukla et al., 2021). This is achieved by (i) the identification (*de novo* or from a literature-based consensus) of a transcriptional signature characterizing the disease [i.e., usually transcripts demonstrated to be up- or downregulated in the selected disease model(s)]; (ii) the parallel acquisition of a drug-perturbed transcriptomic profile in the model(s) of interest, to be representative of the pathways and cell responses elicited in reaction to the specific agent; (iii) inspecting the expression of signature genes upon drug perturbation and the further functional assessment of drug candidates able to reverse the associated pathological signature (Aschenbrenner et al., 2021; Shukla et al., 2021; Knoll et al., 2023). This principle finds its agnostic and fully-computational application in the widespread interrogation of the Connectivity Map (CMap) and the NIH Library of Integrated Network-Based Cellular Signatures (LINCS) databases, together providing perturbed transcriptional profiles for more than 30,000 small molecule compounds screened on mostly cancer cell lines (Lamb et al., 2006; Subramanian et al., 2017; Stathias et al., 2020). Propagating this back to the repurposing of drugs against NDDs, further efforts are required to extend the acquisition of drug-perturbed transcriptomic profiles of relevant cells for NDDs rather than *in vitro* cancer models, which can be only partly suited to efficiently predict drug responses in the CNS/PNS.

Recent examples have seen the signature reversal paradigm applied to drug repurposing against NDDs, e.g., with the identification of bumetanide, a loop-diuretic, as particularly active against effects caused by the APOE4 genotype. This prediction has been confirmed *in vitro* and in mouse models, as well as corroborated by clinical data (Taubes et al., 2021). In another study, authors identified more than 50 candidate drugs starting from available single-nucleus transcriptomics data of human AD brain biopsies combined with GWAS information, leveraging cell-type specific signatures of the disease and an integrated network analysis (Parolo et al., 2023). Other network-based approaches have been recently employed to address drug repurposing in distinct Braak stages of AD pathology, integrating the information from reversal of transcriptomic disease signatures with an additional multi-factorial prioritization, e.g., based on the blood–brain barrier (BBB) permeability of compounds and similarity to agents currently in clinical trials (Savva et al., 2022). More recent avenues point toward the use of artificial intelligence approaches to further optimize the unbiased identification of repurposing candidates, such as DRIAD (Drug Repurposing In AD), a machine learning framework investigating associations between AD Braak stage and specific drug-perturbations in differentiated human neural cell cultures (Rodriguez et al., 2021). Another example is Network topology-based deep learning framework to identify disease-associated genes (NETTAG), which uses aggregated genomics profiles and protein–protein interactome data to infer putative risk genes and drug targets impacted by GWAS loci, then used for the network-based prediction of repurposable candidates (Xu J. et al., 2022). Overall, computational and AI-based approaches have the potential to harmonize and integrate the distinct levels of information useful for drug repurposing, from the inspection of clinical and registry data beyond their actual sharing, e.g., through Swarm Learning (Warnat-Herresthal et al., 2021), its integration with multi-omics profiling and other biophysical characterizations, which can be modeled together in a comprehensive framework for a more efficient identification of drugs against targets in NDDs.

### 3.3 Omics-tailored approaches for the *in vitro* screening of drugs

The experimental screening of chemical agents *in vitro* represents an integral part of the early drug discovery pipeline (Hughes et al., 2011). In the last decades, numerous biochemical and cell-based *in vitro* assays have been developed for the high-throughput investigation of a compounds’ ability to interfere with distinct traits of the pathology underlying NDDs (Aldewachi et al., 2021; Maniam and Maniam, 2024). As concerns bioassays on isolated targets, different miniaturized alternatives have been established for High-Throughput Screening (HTS) of small molecules active in distinct pathways in AD/PD, i.e., UV–Vis, chemiluminescence- or fluorescence-based approaches to screen for binding partners of Aβ isoforms [e.g., ThioflavinT assay (Lee S. et al., 2023)] and tau oligomers, as well as of BACE1 (beta-secretase 1), TDP-43 (Buratti et al., 2013), ApoE (Chen et al., 2012), which among others include Fluorescence Resonance Energy Transfer (FRET) assays [e.g., AlphaScreen (Ren et al., 2013)]. In addition, cell-based high-content imaging screening (HCS) alternatives have been reported for the automated analysis of cell morphology changes and their drug-induced dynamics (Ofengeim et al., 2012), as well as mass-spectrometry (MS)-based HTS methods able to visualize protein aggregation in *ex vivo* drug-exposed brain section (Yoshimi et al., 2015) and other phenotypic options (Maniam and Maniam, 2024). Although such approaches provided the field with novel insights on potential drug candidates and interesting targets for NDDs drug development, they do not offer an holistic overview of cellular responses and phenotypic changes upon perturbation. Instead, they focus on specific drug-target interactions on isolated substrates, or inspect very specific, low-dimensional cellular readouts. These reductionist approaches have limitations when considering the complexity of NDDs, which arise from a variety of pathogenic processes and cell types. In this direction, complementary high-dimensionality omics can be exploited not only as described above, for the evaluation of *in vitro* models and the identification of disease biomarkers and signatures, but even in the experimental screening for novel candidate therapeutics. Indeed, these tools can offer a comprehensive overview of the *in vitro* modeled cellular responses to drug perturbations as well as on the potentially engaged druggable pathways.

New and improved computational approaches are pivotal to identify and rank potential candidates against targets in NDDs, not only fostering drug repurposing, but also suggesting completely new chemical entities through AI-assisted strategies, including modeling of docking properties and molecular dynamics of new candidate drugs (Mock et al., 2023; Sadybekov and Katritch, 2023; Oestreich et al., 2024). Nevertheless, the establishment of robust and reproducible approaches for experimental screening and validation of selected compound libraries remains of key importance for effectively bridging the early drug discovery phase to potential downstream development (Aldewachi et al., 2021). Ideally, for the identification of new targets in NDDs, experimental screenings need to converge with general requirements for drug discovery, such as reproducibility, cost-efficiency as well as scalability and throughput. This can be even enhanced by the use of *in vitro* models with increased controllability and reproducibility, taking into account cellular heterogeneity of the CNS and approximating as much as possible mature human phenotypes (Klimmt et al., 2020).

Combined with increasingly sophisticated *in vitro* models, multi-omics measurements can provide a comprehensive readout of cell-type specific responses to drug or small molecule perturbations (Koromina et al., 2019). This information can be leveraged to better understand pharmacodynamics as well as possible toxicological insights in early *in vitro* models (Nguyen et al., 2022). While changes of transcriptomic signatures can themselves be informative of drug effectiveness, especially in the context of reversal of potentially disease-induced transcriptomic alterations, their combination with other cell-type specific functional readouts (e.g., protein aggregation, cell plasticity or evaluation of metabolic reprogramming) would allow to build models predicting drug sensitivity and favorable drug combinations (Vincent et al., 2022). To close the cycle, omics-based experimental screenings can be leveraged to further inform, scale and optimize the performance of computational approaches to refine the development of new drugs (Zielinski et al., 2021).

As previously mentioned, the widespread use of human iPSCs-based models represents a promising avenue for the *in vitro* modeling of NDDs, which is especially well-suited for early drug screening. Generating libraries of human iPSCs bearing different genetic backgrounds would be important to cover the diverse genetics potentially underlying disease predisposition and would follow the precision medicine paradigm of patient-tailored therapy. In light of the fact that familial occurrence of AD and PD is rare with the majority of cases being sporadic and bearing genetically complex traits, this would be a major endeavor but also a major step towards achieving this goal (Kondo et al., 2017; Okano and Morimoto, 2022). Deep multi-omics phenotyping of drug responses using human iPSCs would allow to account for the wide range of inter-individual susceptibility and disease endophenotypes (Okano and Morimoto, 2022). Along this line, a recent study reported the establishment of a framework combining (i) the identification of repurposed drug hits based on the reversal of an AD gene signature covering severe disease stage, early progression of disease pathology, cognitive decline and animal models, with (ii) an experimental workflow for the transcriptomics-based screening of identified hits on human iPSCs-derived cortical neurons (Williams et al., 2019). The study led to the identification of 51 drugs reversing the AD phenotype *in vitro*, suggesting new avenues in omics-based drug repositioning against NDDs. Another work described a novel network-based drug-screening platform based on the generation of human iPSC-derived cerebral AD organoids (iCOs) and their transcriptional evaluation to guide downstream repurposing (Park et al., 2021). Here, the authors identified interesting candidates by mathematical modeling of a high-content screening (HCS) system based on the use of 1,300 organoids from 11 participants, providing a novel strategy for personalized medicine against NDDs.

Overall, the potential of the use of omics to drive drug discovery from *in silico* generation of hits of potential interest up to the experimental screening and validation of drug candidates is an emerging field with high potential. Despite some valuable examples showing high promise for future developments, these avenues are still underexplored in the context of NDDs and worth more thorough exploration in the near future.

## 4 Practical aspects for the design of omics-based drug screens

**Figure 3:**
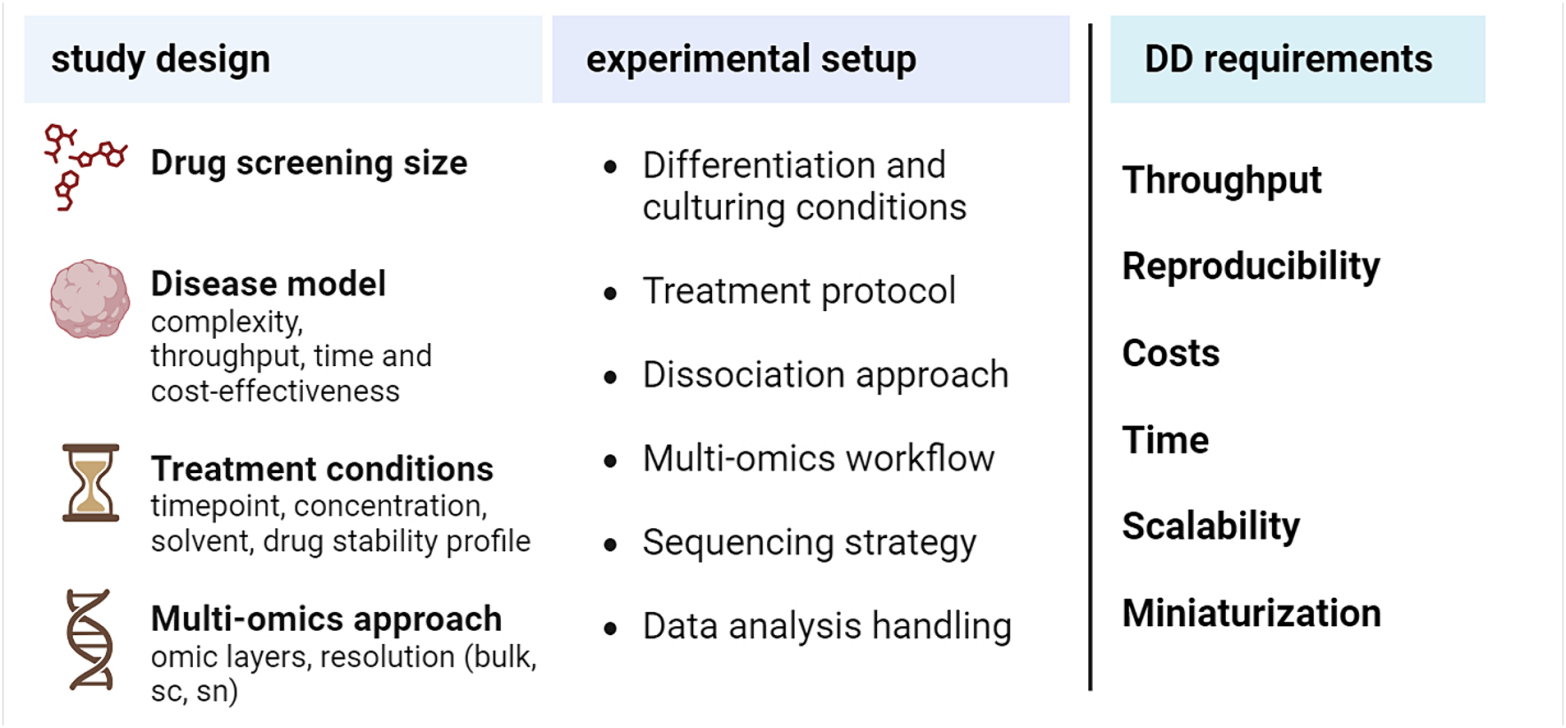
Practical aspects to design an omics-based drug screening experiment. DD, drug discovery, sc, single-cell, sn, single-nucleus. Created with BioRender.com.

In the previous section, we described how multi-omics can offer insights and improve readouts for drug discovery against targets in NDDs. An efficient omics-based drug screening starts from an accurate study design tailored to the biological question of interest (Figure 3). The design should take into account general requirements for drug discovery, such as the need for high reproducibility, which is a prerequisite to subsequently increase the overall throughput, and the possibility for miniaturization, which ensures time- and cost-effectiveness (Aldewachi et al., 2021; Leidner et al., 2024). Together, these two general goals will guarantee a more feasible scale-up of the screening strategy.

Overall, the selection of the right *in vitro* model for the respective NDD is key to the global success of such an endeavor: the choice will depend on a compromise between the desired model complexity (maturation, dimensionality, and heterogeneity, which are maximized in organoid co-cultures), the target throughput and reproducibility, as well as costs and time to be spent on model development and maturation (Whitehouse et al., 2023). While organoids can faithfully reproduce brain development dynamics, they are only partly suited to model NDDs due to their marked embryonic component. In addition, 2D and spheroid-like 3D cultures represent a more valuable alternative also in terms of reproducibility, making them generally more suited to confidently increase the overall throughput (Centeno et al., 2018). An even more promising scenario derives from the combination of human iPSCs-derived neurons and glia with three-dimensional modeling approaches, which allow for the better capture of the complexity of health and disease states even in a heterogeneous milieu (Klimmt et al., 2020). Indeed, co-culturing of neuronal and glial cells would be suited to model the dynamics of the drug response *in vitro* for NDDs reflecting a high degree of cellular complexity and diverse cell–cell communication patterns (Stevenson et al., 2020). Hierarchically increasing the complexity of 3D models, from spheroids to organoids and assembloids, has been demonstrated to enable a more faithful mimicry of intercellular architectures observed in humans, which in analogy should result in a better representation of the dynamics of drug response (Centeno et al., 2018). Nevertheless, higher-order *in vitro* models like organoids still lack sufficient reproducibility and throughput for the required scalability in large drug discovery approaches, while 3D co-culture systems represent a valid compromise between model fidelity and actual throughput (Klimmt et al., 2020). Overall, multi-omics can offer powerful tools to evaluate the fidelity of NDD *in vitro* models and drive the selection of optimal drug screening platforms.

A second key factor is the planned size of the drug screening. An initial high-throughput screening of a larger library of molecules, with omics as primary read-out, would benefit from a miniaturized setting with lower resolution and model complexity, to minimize resources while still maintaining economic feasibility. Instead, the downstream characterization of positive hits would urge for an upgrade of both the *in vitro* model and the omics resolution, so as to enable a further investigation of mechanisms of action and cell-type specificities. In addition, for each of the compounds to be screened, treatment conditions (i.e., time, concentration, solvent for drug solubilization) may need to be optimized *a priori* based on the available literature, or experimentally through multiple screening rounds using proper functional readouts (Moffat et al., 2017). Further, in order to calibrate the experiment, the expected dynamics of time- and dose-dependent responses have to be considered and integrated into the workflow.

Ideally, the most advantageous omic readout layer for screening has to be considered upfront, e.g., genomics, epigenomics, transcriptomics, proteomics, metabolomics. Transcriptome analysis is widely employed to inspect holistic cellular processes at basal or drug-perturbed states and offers the possibility to opt for bulk or single-cell approaches (Hasin et al., 2017). RNA-seq technologies are among the most advanced approaches for further assay miniaturization, and as such bulk and single-cell transcriptomics have been increasingly used in drug discovery in the last decade (Baysoy et al., 2023; Van de Sande et al., 2023). While less easy to interpret as a readout for short-term drug responses, epigenomics approaches have been increasingly reported to assist the investigation of cellular mechanisms of response and drug susceptibility, and an increasing number of technologies are available for their single-cell and spatial characterization (Minnoye et al., 2021; Carraro et al., 2023; Preissl et al., 2023). Proteomics also offers powerful tools for the analysis of general cellular dynamics and response mechanisms, but techniques able to analyze the whole proteome at the resolution of single-cells, especially if starting from a limited number of cells, are still under development (Bennett et al., 2023). Assessing multiple omics layers simultaneously (multi-omics) can provide a more refined picture of drug responses (Carraro et al., 2022; Ivanisevic and Sewduth, 2023). Different strategies have been developed for the proper interpretation of distinct layers of omics information and their cross-talk within the biological context of interest. These integrative approaches, spanning from quantitative causal modeling to latent space inference and late integration (Miao et al., 2021), can tackle challenges such as the handling of heterogeneous data structures and employed technologies (i.e., sequencing- or MS-based), the often non-linear interactions between features from distinct omics readouts, as well as the optimization of computational performance (Athieniti and Spyrou, 2023). Both bulk- or single-cell-tailored strategies have been reported for the efficient integration of multi-omics in NDDs drug and target discovery (Cuperlovic-Culf and Badhwar, 2020; Qiu and Cheng, 2024). As an example, Qiu et al. (2024) recently developed a transcriptomics and proteomics-based systems biology framework to identify AD-related GPCRs and gut metabolites. Further, El Bouhaddani et al. (2024) reported a novel probabilistic multi-omics data integration method combining the information of transcriptomics, proteomics, and drug screening data to identify druggable targets involved in synucleinopathies.

Once the NDD *in vitro* model, the (multi)-omics readout and screening size are defined, additional optimization at different steps should be considered, e.g., related to (i) the general protocol for differentiation and culturing conditions; (ii) the workflow for applying compound treatments, which will vary also based on the planned drug screening size and may call for further miniaturization while maintaining compatibility with the required information depth of the selected omics readout; (iii) the optimal dissociation strategy in case of further cell sorting and single-cell analyses, balancing the overall recovery rate while limiting cellular stress, which could derive from enzymatic trypsin/papain-based digestion with or without manual or automated mechanical dissociation (Denisenko et al., 2020); (iv) the definition of standard operating procedures (SOPs) for the preprocessing of samples for downstream omics investigations (e.g., transcriptomics and/or proteomics; bulk, single-cell or single-nucleus) as well as SOPs for the actual omics analysis based on the specific technology of choice [e.g., droplet-based or microwell-based transcriptomics (Conte et al., 2024)]; (v) the optimal sequencing strategy in case of NGS-based omics, as well as (vi) the downstream analysis workflow based on quality control (QC) requirements, formulated hypotheses to be tested and biological insights to be gained.

In summary, we discussed here some of the main aspects to be considered when planning *a priori* an omics-based drug screening experiment, as well as further steps to be taken into consideration for the optimization of a comprehensive workflow, bearing in mind that every strategy needs to be tailored to the specific biological question, desired throughput and available resources.

## 5 Discussion

The advent of omics technologies has revolutionized the field of health sciences by introducing the paradigm of precision medicine (Ashley, 2016). Precision medicine aims to prevent and treat disease states based on the susceptibilities of individuals through deep geno-phenotyping and further clinical stratification (Hulsen et al., 2019). Such a perspective for personalized therapies sounds particularly appealing to tackle neurodegenerative diseases, which are still rather resistant to a successful discovery of novel curative or symptom-alleviating agents because of their high intra- and inter-individual heterogeneity (Strafella et al., 2018).

In this review, we summarized the main advances and opportunities for the use of multi-omics approaches for drug discovery and development against neurodegenerative diseases. We described (i) how omics can be used to investigate the different cell states, phenotypes and peculiarities of *in vitro* models for NDDs, instructing the downstream selection of the most optimal platforms for drug screening; (ii) how to mine and re-use available omics datasets and databases to refine disease signatures and identify new druggable targets; (iii) how the use of computational omics-based approaches can drive the selection of repurposable candidates including valuable examples from the field; (iv) how the use of omics-based screening approaches can offer a holistic overview of cell type-specific drug responses up to single-cell resolution for an unbiased identification of interesting therapeutics against NDDs and (v) we provided an overview of some practical aspects for the efficient design of an omics-based drug screening experiment.

Among others, these approaches promise to tackle the current lack in early disease intercepting therapies in the field of neurodegeneration, but also highlight the concurrent need for establishing clear blood, CSF, CNS biomarkers as well as more comprehensive definition of patient endotypes across different NDDs to support this process (Hampel et al., 2021). Coupled with high-throughput *in vitro* models for the respective disease, such as hiPSCs-based systems, multi-omics could enable high-scale phenotyping of inter-individual variabilities in favor of more personalized treatments (Brooks et al., 2022). In addition, omics analysis promises to tackle the urgent need to converge and rationalize the information from different valuable rodent and human models, often only partly superimposable, identifying shared and model-specific disease traits, finding univocal cell subtype nomenclatures and prioritize human-like phenotypes and signatures (Aerqin et al., 2022; Jung and Kim, 2023).

Collectively, the advent of omics in driving drug discovery against NDDs has already highlighted promising developments, but further efforts are required to exploit their full potential and increase their clinical translatability. Processing standards and community guidelines would ensure the optimal use of omics in drug development, repurposing and target identification, ranging from sample procurement and preprocessing to multi-omics library generation, data exploration and integration (Mangul et al., 2019; Tarazona et al., 2020; Brooks et al., 2024). In parallel, solid and versatile platforms should be set up for early *in vitro* high-throughput omics screening of drugs against NDDs, guaranteeing reproducibility, scalability and sufficient disease complexity. Moreover, the quality and reusability of multi-omics and clinical data heavily depend on the application of appropriate and harmonized quality assurance protocols for laboratories in the field (Oldoni et al., 2022). Also for AI-based drug discovery, the accuracy of omics-driven data modeling in predicting patient characteristics depends on the quality and quantity of data and the interoperability of the tools used. Reliable and standardized data are not always accessible for AI due to the sensitive nature of clinical phenotype data and challenges in obtaining standardized, structured databases (Oldoni et al., 2022). In addition, data privacy issues pose a significant challenge preventing the full translatability of omics and their use to define reliable biomarkers and drugs (Oestreich et al., 2021). This has an impact on data accessibility and interoperability, and requires the definition of novel strategies beyond classical data sharing practices, able to account for regulatory compliance, ethical and legal considerations (Schultze et al., 2022).

We believe a joint investment in clinical omics-based investigation of disease cohorts together with advanced *in vitro* modeling of NDDs can speed up the identification, further optimization and development of therapeutics to finally intercept, decelerate and potentially cure common neurodegenerative diseases.

## Author contributions

CC: Conceptualization, Data curation, Formal analysis, Investigation, Supervision, Validation, Visualization, Writing – original draft, Writing – review & editing. JM: Formal analysis, Visualization, Writing – original draft, Writing – review & editing. JK: Writing – original draft, Writing – review & editing. DP: Funding acquisition, Supervision, Writing – original draft, Writing – review & editing. JLS: Funding acquisition, Supervision, Writing – original draft, Writing – review & editing. MDB: Conceptualization, Funding acquisition, Project administration, Supervision, Writing – original draft, Writing – review & editing.
